# An Efficient Implementation of Fixed Failure-Rate Ratio Test for GNSS Ambiguity Resolution

**DOI:** 10.3390/s16070945

**Published:** 2016-06-23

**Authors:** Yanqing Hou, Sandra Verhagen, Jie Wu

**Affiliations:** 1 College of Aerospace Science and Engineering, National University of Defense Technology, Deya Road 109, Kaifu District, Changsha 410073, China; Yanqing.Hou@hotmail.com; 2 Faculty of Civil Engineering and Geosciences, Delft University of Technology, Stevinweg 1, Delft 2628 CN, The Netherlands

**Keywords:** GNSS, GPS, ambiguity resolution, ratio test, failure rate, fix rate

## Abstract

Ambiguity Resolution (AR) plays a vital role in precise GNSS positioning. Correctly-fixed integer ambiguities can significantly improve the positioning solution, while incorrectly-fixed integer ambiguities can bring large positioning errors and, therefore, should be avoided. The ratio test is an extensively used test to validate the fixed integer ambiguities. To choose proper critical values of the ratio test, the Fixed Failure-rate Ratio Test (FFRT) has been proposed, which generates critical values according to user-defined tolerable failure rates. This contribution provides easy-to-implement fitting functions to calculate the critical values. With a massive Monte Carlo simulation, the functions for many different tolerable failure rates are provided, which enriches the choices of critical values for users. Moreover, the fitting functions for the fix rate are also provided, which for the first time allows users to evaluate the conditional success rate, i.e., the success rate once the integer candidates are accepted by FFRT. The superiority of FFRT over the traditional ratio test regarding controlling the failure rate and preventing unnecessary false alarms is shown by a simulation and a real data experiment. In the real data experiment with a baseline of 182.7 km, FFRT achieved much higher fix rates (up to 30% higher) and the same level of positioning accuracy from fixed solutions as compared to the traditional critical value.

## 1. Introduction

Precise positioning uses the carrier phase measurements, which inherently contain unknown cycle ambiguities [1]. The high precision is only achievable if the ambiguity is correctly fixed to integers. On the contrary, incorrectly-fixed integer ambiguities may result in large positioning errors. In order to exclude those incorrectly-fixed integer ambiguities, the validation of integer ambiguity is demanded. Integer ambiguity validation has been richly researched, and several methods have been proposed, such as the ratio test [2,3], the difference test [4,5], the projector test [6,7] and the F-test.

Among these methods, the most extensively used one is the ratio test with fixed critical values [6,8,9,10,11]. Verhagen and Teunissen [12] studied the relations between failure rate, false alarm rate and critical values based on the model strength. It was concluded that the traditional ratio test using fixed critical values without distinguishing model strength may either raise false alarm rates or imply no control of the failure rate.

Therefore, the Fixed Failure-rate Ratio Test (FFRT) was proposed, for which critical values dependent on the model strength are selected. The critical value by which the failure rate threshold is fulfilled was shown to be indexed by the number of ambiguities and the Integer Least-Squares (ILS) [13,14,15,16] failure rate. A look-up table of critical values for two typical tolerable failure rates 0.01 and 0.001 is given in [12]. Following the fixed failure-rate idea, Wang and Verhagen [5] studied the Fixed Failure-rate Difference Test (FFDT) and provided the critical values by fitting functions according to the tolerable failure rate and the model strength.

Brack and Günther [17] extends the fixed failure rate idea by proposing a General Integer Aperture (GIA) estimation that tests each entry of the ambiguity vector with critical values calculated from the tolerable failure rate. Supposedly, GIA may accept at least a subset of fixed integer ambiguities rather than occasionally rejecting all.

Compared to the approach using a look-up table [12], this contribution provides fitting functions to describe the relation between the critical value of the ratio test and the model strength even better. A wider range of tolerable failure rates used for FFRT are provided, i.e., ranging from 0.0005 to 0.01, as each user may have their own requirements on the failure rate of Ambiguity Resolution (AR). Additionally, the resulting fix rate is provided by fitting functions, which for the first time will allow users to evaluate the conditional success rate (i.e., the success rate once the integer candidates are accepted by FFRT, see Equation (9)) before AR is performed. The fitting functions for both the critical value of the ratio test and the corresponding fix rate are model dependent, meaning that users can evaluate the possible fix rate and conditional success rate and decide whether AR is worthy or not before the time-consuming AR process. Besides, the functions are easy-to-implement, requiring no efforts to repeat the simulations the authors have done.

This paper is organized as follows. Section 2 briefly reviews the general models and describes the procedure to find the fitting functions of critical values. Section 3 validates the performance of the fitting functions in controlling the failure rate by simulation, and Section 4 shows an example where the FFRT increases the fix rate compared to the conventional ratio test using a real data experiment. Section 5 summarizes the main contributions.

## 2. Methodology

### 2.1. General Ambiguity Resolution Model

A GNSS observation model can be put in the following linearized equation:

(1) $$y=Aa+Bb+e,\;\mathrm{with}\;e∼N(0,Q_{\mathrm{ee}})$$

where $y\in R^{m}$ is the vector of code and carrier observations; $a\in Z^{n}$ is the vector of unknown integer carrier phase ambiguities; $b\in R^{p}$ is the vector of baseline coordinates and may possibly include residual atmospheric delays, as well; $e\in R^{m}$ is the vector of measurement noise, which is assumed to have a zero-mean Gaussian normal distribution; *A* and *B* are the design matrices for the ambiguities and baseline components, respectively; *m*, *n* and *p* are the number of measurements, number of integer ambiguities and number of unknown baseline parameters, respectively.

GNSS precise positioning usually contains four steps [13,14,15]: (1) estimate the float ambiguities and position coordinates; (2) fix float ambiguities to integer values; (3) validate the integer ambiguities; and (4) update the position coordinates using fixed ambiguities.

The float ambiguities and baseline solution can be resolved by weighted least-squares estimation, and their variance covariance (vc) matrix can be obtained using the error propagation law. The float solution and vc-matrix are shown as:

(2) $$\left\{\begin{array}{cc} \left[\begin{array}{c} \hat{a}\\ \hat{b} \end{array}\right] & ={\left({\left[\begin{array}{c} A,B \end{array}\right]}^{T}Q_{\mathrm{ee}}^{-1}\left[\begin{array}{c} A,B \end{array}\right]\right)}^{-1}{\left[\begin{array}{c} A,B \end{array}\right]}^{T}Q_{\mathrm{ee}}^{-1}y\\ \left[\begin{array}{cc} Q_{\hat{a}\hat{a}} & Q_{\hat{a}\hat{b}}\\ Q_{\hat{b}\hat{a}} & Q_{\hat{b}\hat{b}} \end{array}\right] & ={\left({\left[\begin{array}{c} A,B \end{array}\right]}^{T}Q_{\mathrm{ee}}^{-1}\left[\begin{array}{c} A,B \end{array}\right]\right)}^{-1} \end{array}\right.$$

The second step is referred to as Ambiguity Resolution (AR). AR fixes the float ambiguities to integers:

(3) $$\overset{ˇ}{a}=I\left(\hat{a}\right)$$

with $I:R^{n}\mapsto Z^{n}$ the integer mapping from the *n*-dimensional space of real numbers to the *n*-dimensional space of integers. The most extensively-used AR methods are Integer Rounding (IR), Integer Bootstrapping (IB) [18,19] and ILS [13,14]. The mapping function I is different for different AR methods. Due to the discrete nature of $Z^{n}$, I will be a many-to-one map, which means different $\hat{a}$ can be fixed to the same $\overset{ˇ}{a}$. The set of $\hat{a}$ that is mapped by I to the same integer z is defined as the pull-in region of z [20] and can be written as:

(4) $$S_{z}=\left\{x\in R^{n}|z=I\left(x\right),\;z\in Z^{n}\right\}$$

**Figure 1:**
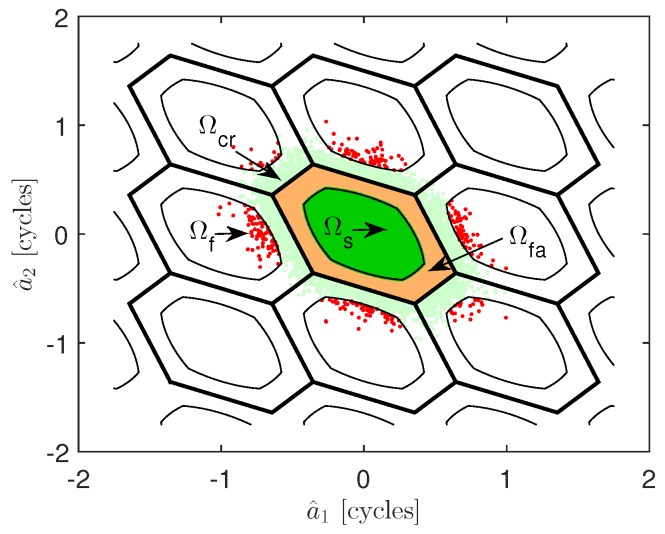
The two-dimensional acceptance region of the Fixed Failure-rate Ratio Test (FFRT). The green and red areas are the regions of correct acceptance and incorrect acceptance. The orange and light green areas are the region of false alarm and correct rejection.

As an example, the pull-in regions of the ILS method for two-dimensional ambiguity vector a are presented by the hexagons in Figure 1. More details of pull-in regions can be found in [20].

The ILS method is efficiently implemented in the LAMBDA software [21]. ILS has the optimal performance regarding the success rate, i.e., the probability of correctly fixing the integer ambiguities [22]. In this study, we use ILS to solve the ambiguities.

The third step validates the fixed ambiguities using an ambiguity acceptance test, for instance the ratio test [2,3], the difference test [4,5], the projector test [6,7] the F-test or the GIA test [17,23]. The most extensively-used test is the ratio test with fixed critical values. Verhagen and Teunissen [12] proposed the Fixed Failure-rate Ratio Test (FFRT), which tunes the critical value to control the failure rate.

The ratio test is given by:

(5) $$\mathrm{Accept}\overset{ˇ}{a}\;\mathrm{if}:\;RT=\frac{\left|\right|\hat{a}-\overset{ˇ}{a}{\left|\right|}_{Q_{\hat{a}\hat{a}}}^{2}}{\left|\right|\hat{a}-{\overset{ˇ}{a}}_{2}{\left|\right|}_{Q_{\hat{a}\hat{a}}}^{2}}<\mu$$

where $\overset{ˇ}{a}$, ${\overset{ˇ}{a}}_{2}$ are the best and second best integer candidates (i.e., the closest and second closest integer vectors to the float ambiguity vector $\hat{a}$, respectively); ${\left||.|\right|}_{Q}^{2}={\left(.\right)}^{T}Q^{-1}\left(.\right)$; *μ* is the critical value of the ratio test.

The ratio test defines aperture pull-in regions, such that the fixed solution $\overset{ˇ}{a}$ is only accepted if the corresponding float ambiguity solution $\hat{a}$ is within this region. The critical value *μ* determines the size of the aperture and thereby determines the probability of incorrect fixing.

A two-dimensional example of the aperture pull-in regions is shown in Figure 1. Since the measurement y is normally distributed, the least-squares estimation $\hat{a},\hat{b}$ from y is also normally distributed. The float ambiguity solution $\hat{a}$ is distributed as:

(6) $$\hat{a}∼N\left(a,Q_{\hat{a}\hat{a}}\right)$$

where the true integer value is $a={\left[0,\;0\right]}^{T}$. The hexagons (solid line) are the ILS pull-in regions, and the aperture pull-in regions (i.e., acceptance regions) are shown, as well. The green and red float samples reside in the acceptance regions $\Omega_{s}$ and $\Omega_{f}$ and are the correctly-fixed and incorrectly-fixed ambiguities, respectively. The remaining regions are the rejection regions $\Omega_{fa}$ and $\Omega_{cr}$, where the orange and light green colors indicate samples that are falsely rejected and correctly rejected, respectively.

### 2.2. Probability Parameters of the Ratio Test

The probability parameters are calculated as the integrals of the Probability Density Function (PDF) of $\hat{a}$ over the regions, as shown in Equation (7).

(7) $$\begin{array}{cc} P_{s} & =\int_{\Omega_{s}}f_{\hat{a}}\left(x\right)dx\\ P_{f} & =\int_{\Omega_{f}}f_{\hat{a}}\left(x\right)dx\\ P_{fa} & =\int_{\Omega_{fa}}f_{\hat{a}}\left(x\right)dx\\ P_{cr} & =\int_{\Omega_{cr}}f_{\hat{a}}\left(x\right)dx \end{array}$$

with the PDF of $\hat{a}$:

(8) $$f_{\hat{a}}\left(x\right)=\frac{1}{\sqrt{det(2\pi Q_{\hat{a}\hat{a}})}}exp\left\{-\frac{1}{2}x^{T}Q_{\hat{a}\hat{a}}^{-1}x\right\}$$

Furthermore, the fix rate and conditional success rate are calculated as follows.

(9) $$\begin{array}{cc} P_{fix} & =P_{s}+P_{f}\\ P_{sf} & =\frac{P_{s}}{P_{fix}}=\frac{P_{fix}-P_{f}}{P_{fix}} \end{array}$$

where the subscript ${\left(.\right)}_{sf}$ denotes successful fixing. The conditional success rate is the success rate conditioned on the integer ambiguities being accepted by FFRT, which indicates the reliability of validated ambiguities. If the failure rate $P_{f}$ is close to zero, this conditional success rate will be close to one. Thus, if the failure rate is small, users can be very confident about the correctness of the integer ambiguities accepted by the ratio test. To evaluate $P_{sf}$, the failure rate and fix rate after FFRT validation $P_{fix}$ are needed.

Due to the complex integration over the aperture pull-in regions of all discrete integer candidates (see Equation (7)), it is impossible to calculate them with analytical formulas [20,21,24]. Therefore, we use Monte Carlo simulation to study the relation between the failure rate, fix rate and the critical value of the ratio test. In total, 25,920 models with different satellite geometries (depending on location and time), GNSS constellations, frequencies, ionospheric and tropospheric delays were simulated, and for each model, $10^{6}$ float solution samples were simulated. The detailed setup is presented in Table 1. The notations $\sigma_{\phi }$ and $\sigma_{\rho }$ represent the standard deviations of undifferenced phase and code measurements in the zenith direction, respectively; $\sigma_{\iota }$ represents the standard deviation of undifferenced ionospheric pseudo measurement in the zenith direction, as is used in the ionospheric-weighted model [25]; el and $P_{f}^{tol}$ represent elevation angle and tolerable failure rate, respectively; the cutoff angle is the elevation mask, such that the satellites with lower elevation angles are not used.

The simulation procedure to obtain proper *μ* and $P_{fix}$ for different tolerable failure rates $P_{f}^{tol}$, ambiguity numbers *n* and ILS failure rates $P_{f,ILS}$ is described in Appendix A.

**Figure 2:**
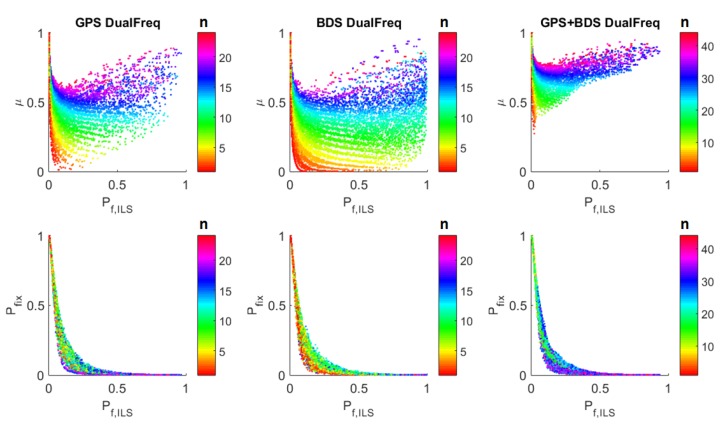
The relation of critical value *μ* and the fix rate $P_{fix}$ against the Integer Least-Squares (ILS) failure rate $P_{f,ILS}$ for the ratio test (see Equation (5)), with tolerable failure rate $P_{f}^{tol}=0.001$. The upper panels show *μ* against $P_{f,ILS}$, and the lower panels show $P_{fix}$ against $P_{f,ILS}$. The color bar indicates the number of ambiguities. The left, middle and right panels show the GPS dual frequency, BDSdual frequency and GPS + BDS dual frequency modes, respectively.

Take $P_{f}^{tol}=0.001$ for an example, the scatter of *μ* against $P_{f,ILS}$ from the simulation is shown in Figure 2. Comparing the upper panels, we can see that there is only ambiguity number differences among these three different constellations considering the relation of *μ* and $P_{f,ILS}$, and the changing trend of the curve for each ambiguity number is not constellation dependent. Therefore, the three constellations are not treated differently in studying the relation of *μ* and $P_{f,ILS}$.

The upper panels show similar results as in [12]:

1. The values of *μ* are grouped by *n*. The more the ambiguities, the larger the value of *μ*.
2. *μ* decreases with the increase of $P_{f,ILS}$ and when the number of ambiguities is large, it later increases again.

The reason for this trend is added in Appendix B.

The lower panels show the relation of $P_{fix}$ against $P_{f,ILS}$ with a fixed $P_{f}^{tol}$. The main findings are:

1. $P_{fix}$ decreases as $P_{f,ILS}$ increases.
2. The values of $P_{fix}$ are grouped by *n*. It does not show the monotonously increasing or decreasing relation with *n*.

### 2.3. Fitting Functions for the Fixed Failure-Rate Ratio Test

We fit *μ* against $P_{f,ILS}$ within a certain range of $P_{f,ILS}$. On the one hand, if $P_{f,ILS}<P_{f}^{tol}$, the best integer candidate is always accepted, and *μ* can be set equal to one. On the other hand, based on the relation between $P_{fix}$ and $P_{f,ILS}$, when $P_{f,ILS}$ is larger than 0.2, the acceptance region will be so small that the fix rate will be low, which has also been mentioned in [12]. Considering this, we select the range as $P_{f}^{tol}\leq P_{f,ILS}<0.2$. In order to get a safe failure rate, we fit the minimum *μ* against $P_{f,ILS}$, which corresponds to the minimum values of *μ* within very small bins (i.e., the bin width is 0.001) over $P_{f,ILS}$. The minimum *μ* and its fitted counterpart will be denoted as $\mu_{min}$ and ${\hat{\mu }}_{min}$, respectively.

Several non-linear functions were tried in the fitting process, including polynomial function series, exponential function series, power function series and rational function series, with the non-linear least-squares method [27]. Among the above function series, four fitting functions were found to perform well:

(10) $$\begin{array}{cc} f_{1}\left(x\right) & =p_{1}x^{p_{2}}\\ f_{2}\left(x\right) & =p_{1}x^{p_{2}}+p_{3}\\ f_{3}\left(x\right) & =p_{1}*e^{p_{2}x}+p_{3}e^{p_{4}}\\ f_{4}\left(x\right) & =\left(p_{1}x^{2}+p_{2}x+p_{3}\right)/\left(x+p_{4}\right) \end{array}$$

judged by the Root Mean Square Error (RMSE):

(11) $$RMSE=\sqrt{\frac{\sum_{i=1}^{n\mu }{\left(\mu_{min}^{i}-{\hat{\mu }}_{min}^{i}\right)}^{2}}{n\mu -np}}$$

where $\mu_{min}^{i}$ and ${\hat{\mu }}_{min}^{i}$ are the *i*-th $\mu_{min}$ and its fitted counterpart through non-linear least squares; nμ and np are the number of $\mu_{min}$ samples and the number of coefficients, respectively. A RMSE value closer to zero indicates a fit that is more useful for prediction. If two or more function candidates obtain a small RMSE, the candidate with fewer coefficients is preferred, since it requires less effort to implement the function.

**Figure 3:**
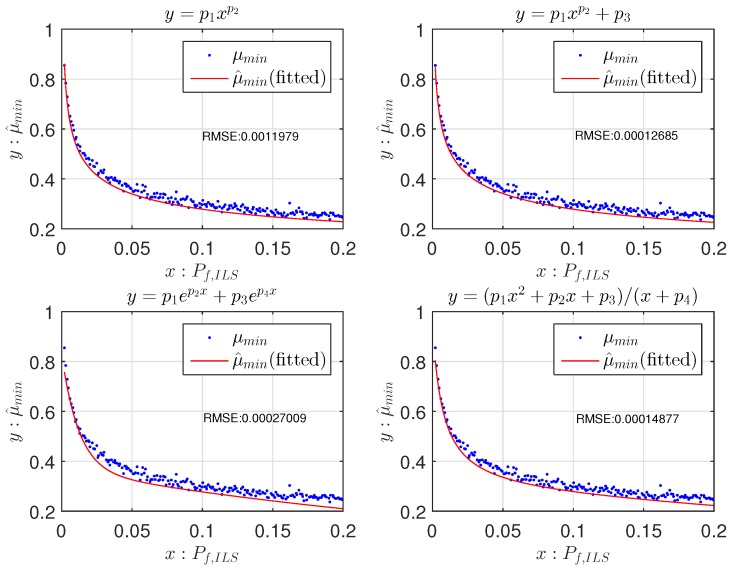
The 95% lower boundary of fitting function candidates of $\mu_{min}$ against $P_{f,ILS}$ and the RMSE (see Equation (11)). The tolerable failure rate $P_{f}^{tol}=0.001$ and the number of ambiguities is eight in this example.

Due to the characteristic of least-squares fitting, there will be both positive and negative fitting residuals, whereas for a safe failure rate, we only accept positive fitting residuals, i.e., the cases where ${\hat{\mu }}_{min}\leq \mu_{min}$. Therefore, the 95% lower boundary of the fitted function is used instead of the original function to prevent negative fitting residuals. Hence, from now on, the 95% lower boundary is referred to as the fitting function. The example in Figure 3 shows the performances of the four fitting function candidates with the number of ambiguities n=8.

**Figure 4:**
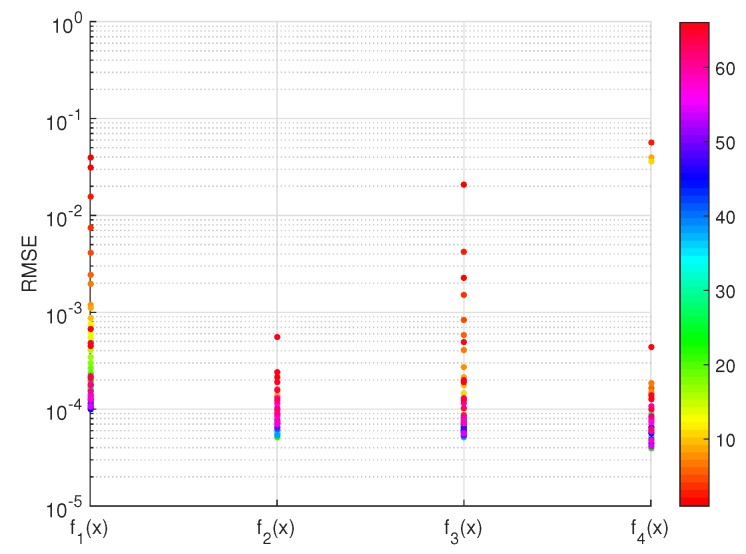
The RMSE of fitting functions $f_{i}\left(x\right),i=1,2,3,4$ for all different numbers of ambiguities *n*. The tolerable failure rate $P_{f}^{tol}=0.001$ and the color bar indexes *n*.

Functions $f_{2}\left(x\right)$ and $f_{4}\left(x\right)$ obtain the smallest RMSE, and $f_{2}\left(x\right)$ has one less parameter. Therefore, $f_{2}\left(x\right)$ is chosen as the best function candidate. For each $f_{i}\left(x\right),i=1,2,3,4$, the RMSEs of all different numbers of ambiguities *n* are shown as dots in Figure 4. $f_{2}\left(x\right)$ and $f_{4}\left(x\right)$ obtain the lowest RMSEs in most cases, ranging around $10^{-4}$; and $f_{2}\left(x\right)$ has one parameter less than $f_{4}\left(x\right)$.

Thus, the fitting function of *μ* is generally chosen as:

(12) $$f_{\mu }\left(x\right)=p_{1}x^{p_{2}}+p_{3}$$

The full table of coefficients for all $P_{f}^{tol}$ in Table 1 can be found in the Electronic Supplementary Material (ESM). As an example, the tables of the coefficients for $P_{f}^{tol}=0.01$ and $P_{f}^{tol}=0.001$ are given in the Appendix C.1. The complete function of *μ* against $P_{f}^{tol}$ for each *n* is as follows.

(13) $$\mu =\left\{\begin{array}{cc} 0, & \;P_{f,ILS}\geq 0.2\\ f_{\mu }\left(P_{f,ILS}\right), & \;P_{f}^{tol}\leq P_{f,ILS}<0.2\\ 1, & \;\mathrm{otherwise} \end{array}\right.$$

Additionally, the range of *μ* should be [0,1]. If $f_{\mu }\left(P_{f,ILS}\right)>1$, it is set to one.

Similarly, we fit the resulting $P_{fix}$ from $\mu_{min}$ against $P_{f,ILS}$. The range of $P_{f,ILS}$ is also $P_{f,tol}\leq P_{f,ILS}<0.2$. The polynomial function series, exponential function series, power function series and rational function series were tried, among which the best choice switches between two functions for different numbers of ambiguities *n*, in favor of the smallest fitting residuals and then the fewest coefficients:

(14) $$f_{fix}\left(x\right)=\left\{\begin{array}{cc} q_{1}x^{3}+q_{2}x^{2}+q_{3}x+q_{4}, & \;n=1\\ \frac{q_{1}}{x^{2}+q_{2}x+q_{3}}, & \;\mathrm{otherwise} \end{array}\right.$$

**Figure 5:**
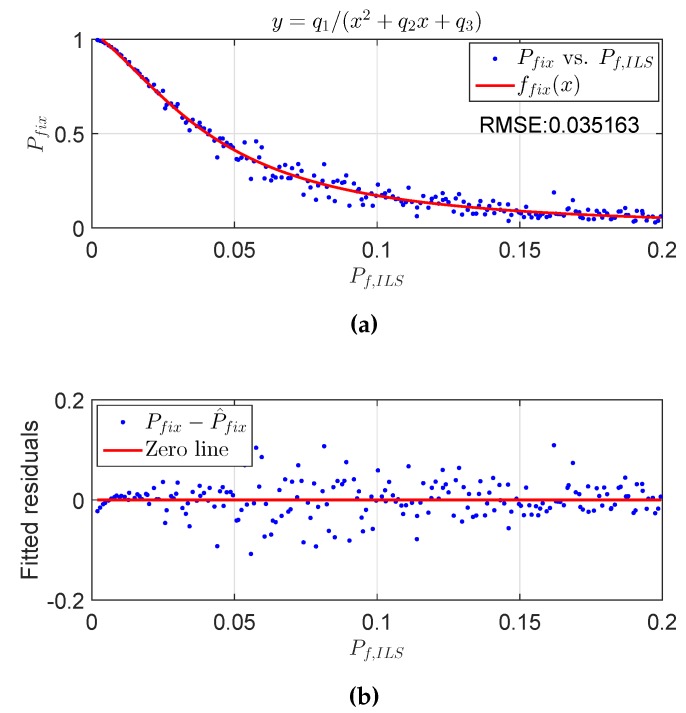
The fitting function of $P_{fix}$ against $P_{f,ILS}$ and its fitted residuals. $P_{fix}$ is resulted from $\mu_{min}$. The upper panel shows the fitted curve, and the lower panel shows the fitted residuals. The tolerable failure rate $P_{f}^{tol}=0.001$, and the number of ambiguities is eight. (**a**) ${\hat{P}}_{fix}$ vs. $P_{f,ILS}$; (**b**) $P_{fix}-{\hat{P}}_{fix}$ vs. $P_{f,ILS}$.

An example of the fitted curve is shown in Figure 5.

The full table of coefficients of $P_{fix}\left(x\right)$ for all $P_{f}^{tol}$ in Table 1 can be found in the ESM. As an example, the tables when $P_{f}^{tol}=0.001$ and $P_{f}^{tol}=0.01$ are shown in the Appendix C.2. The complete function of the $P_{fix}$ against $P_{f,ILS}$ is as follows.

(15) $$P_{fix}=\left\{\begin{array}{cc} 0, & \;P_{f,ILS}\geq 0.2\\ f_{fix}\left(P_{f,ILS}\right), & \;P_{f}^{tol}\leq P_{f,ILS}<0.2\\ 1, & \;\mathrm{otherwise} \end{array}\right.$$

Additionally, the range of $f_{fix}$ should be [0,1]. If $f_{fix}\left(P_{f,ILS}\right)>1$, it is set to one; and if $f_{fix}\left(P_{f,ILS}\right)<0$, it is set to zero.

Note that for *μ*, a rigid conservative fitting is necessary; therefore, the fitted curve is chosen to be lower than most of the $\mu_{min}$; while for $P_{fix}$ an approximation is enough; therefore, the fitted least-squares curve is used. However, since the $P_{fix}$ resulting from $\mu_{min}$ are used in the fitting process, the fitting function of $P_{fix}$ is also conservative.

## 3. Numerical Validation

To show the performance of the fitted *μ* and $P_{fix}$, we did a simulation with all of the models listed in Table 1 and compared the failure rate, false alarm and fix rate with other validation methods. For convenience, we denote *μ* from different methods as in Table 2.

**Figure 6:**
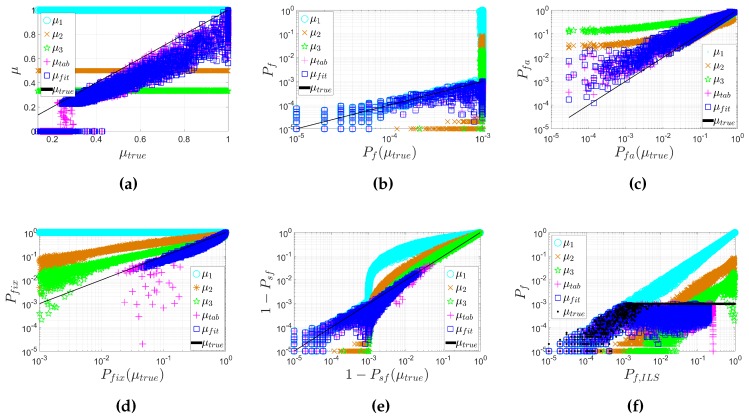
The comparison of critical value *μ*, failure rate $P_{f}$, false alarm rate $P_{fa}$, fix rate $P_{fix}$ and conditional failure rate $\left(1-P_{sf}\right)$ from different methods, with $P_{f}^{tol}=0.001$ and n=8. (**a**) *μ* vs. $\mu_{true}$; (**b**) $P_{f}$ vs. $P_{f}\left(\mu_{true}\right)$; (**c**) $P_{fa}$ vs. $P_{fa}\left(\mu_{true}\right)$; (**d**) $P_{fix}$ vs. $P_{fix}\left(\mu_{true}\right)$; (**e**) $\left(1-P_{sf}\right)$ vs. $\left[1-P_{sf}\left(\mu_{true}\right)\right]$; (**f**) $P_{f}$ vs. $P_{f,ILS}$.

Figure 6 shows the values of *μ* from different methods for all simulated models with n=8 and $P_{f}^{tol}=0.001$, as well as the resulting failure rate, false alarm rate, fix rate and conditional failure rate, i.e., the failure rate once integer ambiguities are accepted by the ratio test. Note that $\mu_{true}$ is the benchmark critical value that exactly controls the tolerable failure rate, i.e., the dots in the upper panels in Figure 2.

In Figure 6a–e, the horizontal axis represents $\mu_{true}$ and its corresponding probability parameters; the vertical axis shows all other *μ* and corresponding probability parameters. In Figure 6f, the horizontal axis represents $P_{f,ILS}$, and the vertical axis shows the corresponding $P_{f}$ for different *μ*.

Figure 6a shows that $\mu_{tab}$ and $\mu_{fit}$ follow the trend of $\mu_{true}$ and are in fact slightly lower, as desired.

Figure 6f shows that only if $P_{f,ILS}<10^{-3}$ (i.e., $P_{f}^{tol}$) the failure rate with $\mu_{true}$ will be lower than $10^{-3}$, while in all other cases, it is very close to $10^{-3}$. The failure rates with $\mu_{tab}$ and $\mu_{fit}$ are always lower than $10^{-3}$, while with $\mu_{2}$ and $\mu_{3}$ are mostly much larger than $10^{-3}$, which is undesirable.

Figure 6b provides deeper insight into the $P_{f}$ with different *μ*. When $P_{f}\left(\mu_{true}\right)<10^{-3}$ (i.e., $P_{f,ILS}<10^{-3}$), $P_{f}\left(\mu_{tab}\right)$ and $P_{f}\left(\mu_{fit}\right)$ slightly vary around $P_{f}\left(\mu_{true}\right)$, but are always lower than $10^{-3}$, while $P_{f}\left(\mu_{2}\right)$ and $P_{f}\left(\mu_{3}\right)$ are much lower than $P_{f}\left(\mu_{true}\right)$ traded-off by many unnecessary false alarms. When $P_{f}\left(\mu_{true}\right)$ is very close to $10^{-3}$, $P_{f}\left(\mu_{tab}\right)$ and $P_{f}\left(\mu_{fit}\right)$ are always lower than $P_{f}\left(\mu_{true}\right)$, while $P_{f}\left(\mu_{2}\right)$ and $P_{f}\left(\mu_{3}\right)$ are in many cases much larger than $P_{f}\left(\mu_{true}\right)$.

Figure 6c shows that the false alarm rates with all choices of the critical value will be larger than with the benchmark result, but the false alarm rates with $\mu_{tab}$ and $\mu_{fit}$ are lower than with $\mu_{2}$ and $\mu_{3}$. Note that when $P_{fa}\left(\mu_{true}\right)$ is close to one, $P_{fa}\left(\mu_{2}\right)$ and $P_{fa}\left(\mu_{3}\right)$ are lower than $P_{fa}\left(\mu_{true}\right)$ due to the lenient critical values, which also cause high failure rates.

Figure 6d shows that the fix rates with $\mu_{tab}$ and $\mu_{fit}$ are slightly lower than the benchmark result, while with $\mu_{2}$ and $\mu_{3}$, the fix rates are much higher than the benchmark result, mainly due to the high failure rates.

Figure 6e shows the conditional failure rate $1-P_{sf}$. It can be seen that when the conditional failure rate with $\mu_{true}$ is lower than $10^{-3}$, the performance with different *μ* is similar as with $P_{f}$ in Figure 6b, since the fix rate is close to one due to very small $P_{f,ILS}$. In all other cases, however, the conditional failure rates with $\mu_{tab}$ and $\mu_{fit}$ are slightly lower than with $\mu_{true}$, while with $\mu_{2}$ and $\mu_{3}$, they are much higher than with $\mu_{true}$.

The results with different ambiguity numbers are similar to the result as shown in Figure 6. Those results are not shown here due to space limits and are given in the ESM of this paper.

Table 3 shows the percentage of the models where $P_{f}$ is controlled below $P_{f}^{tol}$ among all of the simulated models. $\mu_{tab}$ and $\mu_{fit}$ controlled the failure rate for 99.9% and 100% of all of the models, while $\mu_{2}$ and $\mu_{3}$ controlled the failure rate for only 33.7% and 50.2% of all of the models. The difference of performance between $\mu_{fit}$ and $\mu_{tab}$ is slight. The slight difference of percentages between $\mu_{fit}$ and $\mu_{tab}$ is because $\mu_{fit}$ is more conservative than $\mu_{tab}$, since:

1. In the look-up table algorithm, the lowest values are chosen to be *μ* [12], while in the fitting function algorithm, the 95% lower boundary of the original curve fitted from the lowest values is chosen as the final fitting function of *μ*;
2. In the look-up table, *μ* is set to zero when $P_{f,ILS}\geq 0.25$ [12], while in the fitting function, *μ* is set to zero when $P_{f,ILS}\geq 0.20$.

The comparison in Figure 6 and Table 3 indicates that FFRT should be used instead of constant critical values.

## 4. Experiment Validation

To compare the performance of FFRT with respect to the traditional ratio test in real data cases, one week of GPS dual-frequency data in a long baseline (182.7 km) is collected and processed with modified RTKLIB [11,28] software. The experimental setup is shown in Table 4.

In the data processing, the dual-frequency code and phase measurements are used. The ionosphere weighted model [25] is considered and the Zenith Troposphere Delay (ZTD) is estimated every epoch. The rover position is considered as kinematic, and the ambiguities are considered as constant, i.e., the float ambiguities in each epoch are estimated using all of the data from the previous epochs. The integer ambiguities are resolved in each epoch, and the LAMBDA [13,14,16,21] algorithm is used to resolve them. For more details of the model and algorithm, see Appendix E in the RTKLIB manual [28].

**Figure 7:**
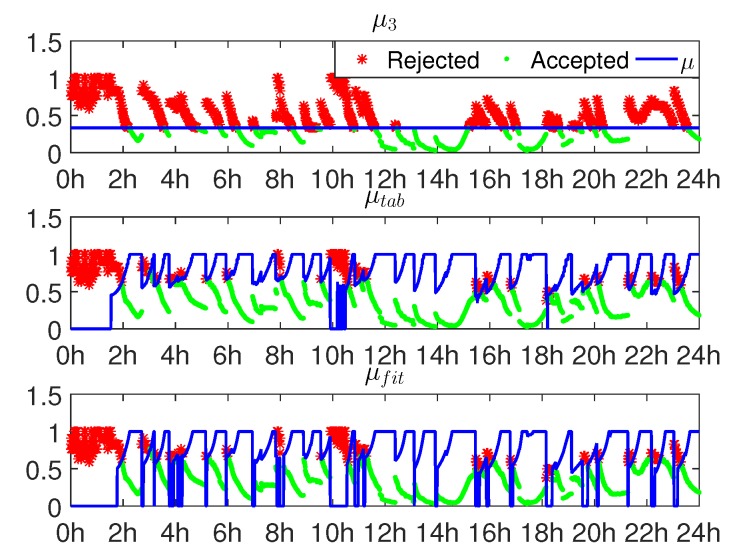
The ratio of AR and *μ* values in one day. The upper, middle and lower panels relate to $\mu_{3}$, $\mu_{tab}$ and $\mu_{fit}$, with $P_{f}^{tol}=0.01$.

Figure 7 shows the ratios and the three thresholds $\mu_{3}$, $\mu_{tab}$ and $\mu_{fit}$ for one day. The tolerable failure rate for $\mu_{tab}$ and $\mu_{fit}$ is $P_{f}^{tol}=0.01$. As seen, to control the failure rate under $P_{f}^{tol}$, both $\mu_{tab}$ and $\mu_{fit}$ vary in different epochs as $P_{f,ILS}$ varies in different epochs.

Since the truth of the ambiguities is unknown, we cannot evaluate the failure rate and success rate of the ambiguity resolution. Instead, to compare the performance with different *μ*, we show the positioning errors of the ratio test-accepted fixed solutions and the fix rates achieved by different *μ*. The positioning errors are calculated as the difference to the true baseline coordinates, where the long-term average coordinates of these two stations from EUREF [29] are used as the true coordinates.

**Figure 8:**
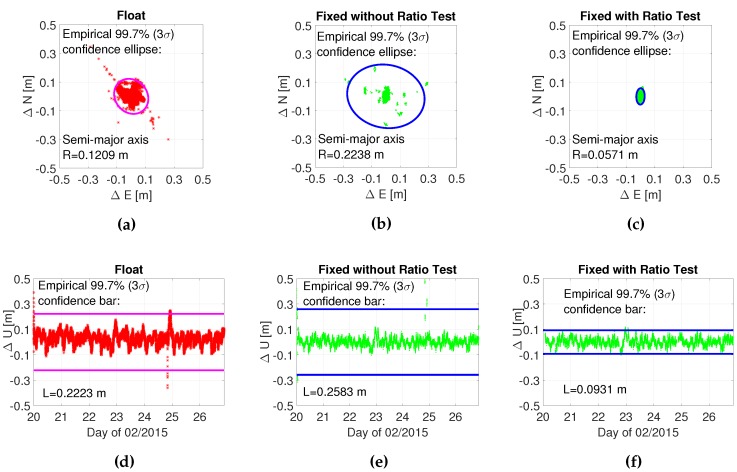
The positioning errors and the 3σ confidence circle (bar) in the horizontal and vertical directions. The left, middle and right panels show float, ILS fixed and ILS fixed with ratio test solutions. The horizontal axis in the lower panels represent the day of 02/2015. (**a**) Float N-E; (**b**) Fixed N-E without the ratio test; (**c**) Fixed N-E with the ratio test; (**d**) Float U-T; (**e**) Fixed U-T without the ratio test; (**f**) Fixed U-T with the ratio test.

Figure 8 shows the positioning errors and empirical 3σ confidence region in the horizontal and vertical directions. The upper and lower panels show the horizontal and vertical errors, and the left, middle and right panels show the results of float, fixed without ratio test and fixed with ratio test solutions. The positioning errors of fixed solutions with different *μ* for the ratio test are very similar; hence, we do not distinguish them here. As seen, the fixed solutions with the ratio test has the smallest confidence ellipse (semi-major axis = 5.71 cm) in the horizontal direction, followed by the float solution (semi-major axis = 12.09 cm) and the fixed solution without ratio test (semi-major axis = 22.38 cm). The performance in the vertical direction is similar. It is clear that the ratio test effectively prevents the incorrectly-fixed ambiguities.

Table 5 shows the fix rates, the probability of positioning errors ϵ≤0.03 m, ϵ≥0.10 m, and ϵ≥0.3 m, for different *μ* and the float solution. In this contribution, 0.03 m is used as the criteria of centimeter accuracy, while 0.10 m is used as the criteria of sub-meter accuracy, and 0.3 m is used as the criteria of large positioning errors caused by wrong fixing. As seen, $\mu_{tab}$ and $\mu_{fit}$ achieve much higher fix rates than $\mu_{2}$ and $\mu_{3}$ (around 30%) and achieve a higher probability of ϵ≤0.03 m. The probability of ϵ≥0.10 m for all $\mu_{2}$, $\mu_{3}$, $\mu_{tab}$ and $\mu_{fit}$ is below 0.01, while $\mu_{tab}$ and $\mu_{fit}$ achieve much higher fix rates. This indicates that $\mu_{tab}$ and $\mu_{fit}$ prevent unnecessary false alarms raised by $\mu_{2}$ and $\mu_{3}$ in this experiment. The probability of ϵ≥0.3 m for all $\mu_{2}$, $\mu_{3}$, $\mu_{tab}$ and $\mu_{fit}$ is 0.0002, while for $\mu_{1}$ is 0.0015. This indicates that although $\mu_{tab}$ and $\mu_{fit}$ achieve high fix rates, it does not result in large positioning errors as $\mu_{1}$ may do.

**Figure 9:**
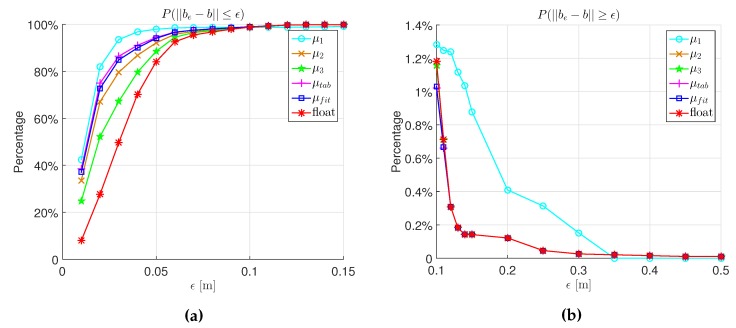
The probability of $\left|\right|b_{e}-b\left|\right|\leq \epsilon$ and $\left|\right|b_{e}-b\left|\right|\geq \epsilon$ for the fixed solution with different *μ* and the float solution. (**a**) $P(||b_{e}-b||\leq \epsilon )$; (**b**) $P(||b_{e}-b||\geq \epsilon )$.

Figure 9 shows the probability $P(||b_{e}-b||\leq \epsilon )$ and $P(||b_{e}-b||\geq \epsilon )$ for different *ϵ*. As seen, although $\mu_{1}$ achieves the highest probability of $\left|\right|b_{e}-b\left|\right|\leq \epsilon$, it also brings many large errors, which is unacceptable. Except $\mu_{1}$, the highest probability of $\left|\right|b_{e}-b\left|\right|\leq \epsilon$ is achieved by $\mu_{tab}$ and $\mu_{fit}$. In the meantime, $\mu_{tab}$ and $\mu_{fit}$ achieve a low probability of $\left|\right|b_{e}-b\left|\right|\geq \epsilon$, as well.

From this real data experiment, we see that the fixed solution with ratio test has the highest accuracy, and compared to the constant *μ* values, $\mu_{tab}$ and $\mu_{fit}$ significantly improve the fix rate without bringing large errors. Therefore, FFRT should always be used instead of the ratio test with constant critical values.

## 5. Conclusions

In this study, we proposed and implemented fitting functions to calculate the critical values of the ratio test according to the required failure rate and number of ambiguities. The functions of *μ* and $P_{fix}$ for different $P_{f}^{tol}$ and different *n* are provided. One example with $P_{f}^{tol}=0.001$ and n=8 is given to show the performance of the new method. Compared to the commonly-used constant critical values, the fixed failure-rate ratio test provided variable critical values according to the model strength, resulting in lower false alarms for strong models and controlled failure rates for weak models. The fitting function method provides more choices of tolerable failure rate $P_{f}^{tol}$ and more *n* than the critical value table. Additionally, the fitting function to compute an approximate fix rate is also provided.

The processing of a 182.7-km baseline real data experiment shows that FFRT improves the fix rate without bringing large positioning errors compared to the ratio test with constant critical values. With the high accuracy of the ratio test accepted fixed solution, this means the improvement of availability. For the above reasons, FFRT is to be preferred above the ratio test with constant critical values.

In this experiment, FFRT contributes to the improvement of accuracy mainly because it avoids unnecessary false alarms. To demonstrate the advantages of FFRT against the traditional ratio test from different aspects, more real data experiment will be done, and the performances will be compared in the future work.

## Figures and Tables

**Table 1:** The setup of the Monte Carlo simulations. ZTD, Zenith Troposphere Delay.

Date	22 November 2013, 23 November 2013, 0:1:23 h (in total 48 epochs)
Location([Lat, Lon])	[30N°, 115E°], [50N°, 115E°], [30N°, 140E°]
Measurements	L1, B1 , L1B1, L1L2, B1B2, L1L2 + B1B2, B1B2B3, L1L2L5, B1B2B3 + L1L2L5
$\sigma_{\phi }$	{2, 3} mm
$\sigma_{\rho }$	{100, 150} × $\sigma_{\phi }$
$\sigma_{\iota }$	{5, 10, 15, 20, 30} mm
Troposphere model	Canceled when $\sigma_{\iota }=5$ mm, and Estimate ZTD when $\sigma_{\iota }>5$ mm
Ionosphere model	Ionospheric weighted model [25]
Elevation ( el) weight	$\sigma^{2}\left(el\right)=\sigma^{2}w\left(el\right)$, $\sigma =\sigma_{\phi },\sigma_{\rho },\sigma_{\iota }$ w(el)=1+10×exp(−el/10) [26]
Cutoff angle	10°
$P_{f}^{tol}$	{5:1:9} $\times 10^{-4}$, {1:1:10} $\times 10^{-3}$

**Table 2:** The notation of *μ* from different methods.

*μ*	Meaning
$\mu_{1}=1$	Accept all candidates.
$\mu_{2}=1/2$	Commonly-used value [6,8,9].
$\mu_{3}=1/3$	Commonly-used value [10,11].
$\mu_{tab}$	From the look-up table [12].
$\mu_{fit}$	Calculated by the fitting function.
$\mu_{true}$	Benchmark value from simulation.

**Table 3:** The percentage of $P_{f}$ being controlled below $P_{f}^{tol}$ by critical values from different methods.

	$\mu_{1}$	$\mu_{2}$	$\mu_{3}$	$\mu_{tab}$	$\mu_{fit}$	$\mu_{true}$
$P(P_{f}<P_{f}^{tol})$	17.9	33.7	50.2	99.9	100	100

**Table 4:** The setup of the real data experiment. AR, Ambiguity Resolution.

Parameter	Value
Time	20 February 2015–26 February 2015 (7 days, 20,160 epochs)
Baseline	WSRA-DLF1(182.7 km)
Measurements	L1L2 code and phase
Cutoff angle	10°
Epoch interval	30 s
$\sigma_{\phi }$	3 mm
$\sigma_{\rho }$	30 cm
$\sigma_{\iota }$	2 cm
Troposphere model	Estimate ZTD
Ionosphere model	Ionosphere-weighted [25]
Elevation (el) weight	$\sigma^{2}\left(el\right)=\sigma^{2}w\left(el\right)$, $\sigma =\sigma_{\phi },\sigma_{\rho },\sigma_{\iota }$ $w\left(el\right)=1+\frac{64}{9sin(el)}$ [28]
Process mode	Kinematic
AR mode	Continuous AR

**Table 5:** Probability parameters. $P_{f}^{tol}=0.01$ for $\mu_{tab}$ and $\mu_{fit}$. $b_{e}$ denotes the estimated baseline solutions, and the subscript ${\left(.\right)}_{e}$ represents estimation.

	$\mu_{1}$	$\mu_{2}$	$\mu_{3}$	$\mu_{tab}$	$\mu_{fit}$	Float
$P_{fix}$	1	0.7732	0.5462	0.8715	0.8241	0
$P(||b_{e}-b||\leq 0.03$ m)	0.9353	0.7961	0.6719	0.8641	0.8487	0.4962
$P(||b_{e}-b||\geq 0.10$ m)	0.0125	0.0071	0.0071	0.0066	0.0067	0.0071
$P(||b_{e}-b||\geq 0.3$ m)	0.0015	0.0002	0.0002	0.0002	0.0002	0.0002

**Figure B1:**
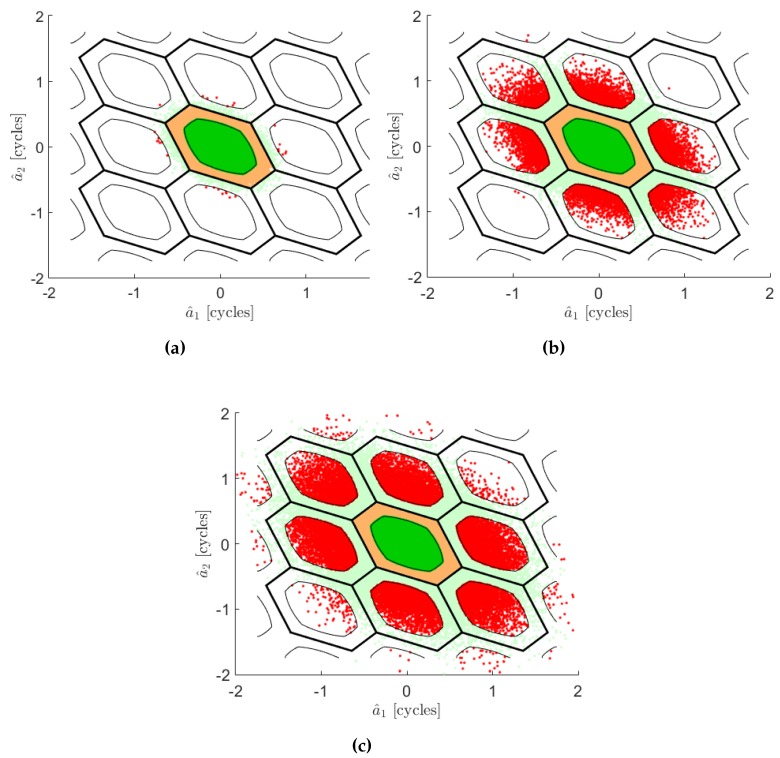
The spread of float ambiguities with $P_{f,ILS}$ increases. (**a**) Low $P_{f,ILS}$; (**b**) Higher $P_{f,ILS}$; (**c**) Even higher $P_{f,ILS}$.

## Supplementary Materials

The following are available online at http://www.mdpi.com/1424-8220/16/7/945/s1: tables of the fitting function coefficient for *μ*: CoefficientMu.csv; tables of the fitting function coefficient for $P_{fix}$: CoefficientPfix.csv; probability parameters of different *μ* with a greater number of ambiguities: FigmoreN.zip.

## Appendix A. Generate the Critical Value of the Ratio Test and the Fix Rate

The simulation is to generate the failure rate and fix rate, with given critical values of the ratio test. Then, the critical value *μ* with a tolerable failure rate $P_{f}^{tol}$ is given. Moreover, the fix rate resulting from this *μ* is given. The Monte Carlo simulation steps are as follows.

1. Generate many different models with various satellite geometries (system, time and location), number of frequencies, measurement noise, baseline length and the accuracy of atmospheric corrections.
2. For each model, calculate $Q_{\hat{a}\hat{a}}$ following the error propagation law, and generate *N* samples $\hat{a}$ with the zero mean and variance $Q_{\hat{a}\hat{a}}$.
3. For each sample ${\hat{a}}_{i}$, Z-transform ${\hat{a}}_{i}$ to ${\hat{z}}_{i}$, and search the best and second best integer candidate of ${\overset{ˇ}{z}}_{o,i}$ and ${\overset{ˇ}{z}}_{s,i}$ with LAMBDA. Calculate the ratio $R_{i}=\frac{\left|\right|{\hat{z}}_{o,i}-{\overset{ˇ}{z}}_{o,i}{\left|\right|}_{Q_{\hat{z}\hat{z}}}^{2}}{\left|\right|{\hat{z}}_{o,i}-{\overset{ˇ}{z}}_{s,i}{\left|\right|}_{Q_{\hat{z}\hat{z}}}^{2}}$.
4. For each $\mu_{j}\in \left[0.001,0.002,...1\right]$, calculate:
  (A1) $$\omega \left(R_{i},\mu_{j}\right)=\left\{\begin{array}{cc} 1, & \mathrm{if}\;R_{i}\leq \mu_{j}\;\mathrm{and}\;{\overset{ˇ}{z}}_{o,i}=0\\ 0, & otherwise \end{array}\right.$$
  and:
  (A2) $$\eta \left(R_{i},\mu_{j}\right)=\left\{\begin{array}{cc} 1, & \mathrm{if}\;R_{i}\leq \mu_{j}\\ 0, & otherwise \end{array}\right.$$
5. After all *N* samples of a specific model are processed in the above three steps, the failure rate and fix rate for each $\mu_{j}$ are calculated as:
  (A3) $$P_{f}\left(\mu_{j}\right)=\frac{N_{f}\left(\mu_{j}\right)}{N},\;P_{fix}\left(\mu_{j}\right)=\frac{N_{fix}\left(\mu_{j}\right)}{N}$$
  with:
  (A4) $$N_{f}\left(\mu_{j}\right)=\sum_{i=1}^{N}\omega (R_{i},\mu_{j}),\;N_{fix}\left(\mu_{j}\right)=\sum_{i=1}^{N}\eta (R_{i},\mu_{j})$$
  Specifically, when $\mu_{j}=1$, $P_{f}\left(\mu_{j}\right)$ is the ILS failure rate $P_{f,ILS}$.
6. The maximum $\mu_{j}$ that meets $P_{f}\left(\mu_{j}\right)\leq P_{f}^{tol}$ is set as the $\mu (P_{f}^{tol})$ for this model.
7. Find $\mu (P_{f}^{tol})$ for all generated models and different $P_{f}^{tol}$.

## Appendix B. The Conceptual Explanation of the Trend of *μ* against the $P_{f,ILS}$ Curve

Starting from a relatively low $P_{f,ILS}$, an increase of $P_{f,ILS}$ will result in a PDF with a larger spread, and as a consequence, more samples will fall in the acceptance regions of incorrect integers, comparing Figure B1a and Figure B1b. Therefore, the *μ* should be decreased with increasing $P_{f,ILS}$.

As the $P_{f,ILS}$ further increases, the PDF of float ambiguities will extend to the ILS pull-in regions centered at even more incorrect integer candidates, i.e., not only the adjacent integers of the true integer. With the *μ* value unchanged, more samples will fall in the rejection regions (i.e., the orange and light green colored regions), and fewer samples will fall in the acceptance regions centered at the adjacent integers (i.e., the density of samples is diluted), which brings a decrease of the failure rate . Meanwhile, the samples that fall in the acceptance region centered at non-adjacent integers result in an increase of the failure rate (but less significantly than the previously-mentioned decreasing effect). The sum of these two effects together slows down the decrease of the *μ* value.

The larger the $P_{f,ILS}$, the slower the *μ* decreases, as seen in Figure 2. Specifically, for high dimensions (i.e., more ambiguities), the ratio of the acceptance region’s volume against the ILS pull-in region’s volume is very small even if *μ* is large. For instance, a scale of edge length *s* (0<s<1) will lead to the scale of $s^{n}$ in the n -dimensional hypercube volume [30]. As a result, the spread of the float ambiguity samples will result in a very significant decreasing effect and a very insignificant increasing effect of the failure rate. If *μ* is unchanged, the failure rate (and the fix rate) will become lower with increasing $P_{f,ILS}$. Therefore, when the ambiguity number is large and $P_{f,ILS}$ is relatively large, with increasing $P_{f,ILS}$, *μ* can be larger to still keep the failure rate at the required value and not lower.

## Appendix C. The Coefficient Table for Fitting Functions

### Appendix C.1. Coefficient Table for Fitting Functions of μ against $P_{f}^{tol}$ in the Ratio Test

**Table C1:** The coefficients for the fitting function of *μ* against $P_{f,ILS}$: $f_{\mu }\left(x\right)=p_{1}x^{p_{2}}+p_{3}$. The tolerable failure rate is $P_{f}^{tol}=0.01$.

*n*	$p_{1}$	$p_{2}$	$p_{3}$	*n*	$p_{1}$	$p_{2}$	$p_{3}$	*n*	$p_{1}$	$p_{2}$	$p_{3}$
1	0.0916	−0.5801	−0.2850	23	0.0514	−0.4286	0.6342	45	0.0249	−0.4505	0.8036
2	0.1576	−0.4633	−0.3145	24	0.0519	−0.4202	0.6435	46	0.0269	−0.4332	0.8037
3	0.2164	−0.3864	−0.2878	25	0.0529	−0.4098	0.6531	47	0.0237	−0.4527	0.8119
4	0.3364	−0.2968	−0.3335	26	0.0425	−0.4442	0.6762	48	0.0250	−0.4390	0.8129
5	0.4401	−0.2435	−0.3686	27	0.0381	−0.4575	0.6916	49	0.0255	−0.4322	0.8148
6	0.3794	−0.2521	−0.2291	28	0.0458	−0.4183	0.6885	50	0.0259	−0.4265	0.8167
7	0.2904	−0.2793	−0.0730	29	0.0386	−0.4443	0.7059	51	0.0231	−0.4418	0.8240
8	0.2874	−0.2702	−0.0146	30	0.0387	−0.4380	0.7124	52	0.0217	−0.4504	0.8280
9	0.1797	−0.3314	0.1593	31	0.0385	−0.4329	0.7204	53	0.0220	−0.4457	0.8305
10	0.1569	−0.3439	0.2290	32	0.0384	−0.4287	0.7267	54	0.0253	−0.4180	0.8279
11	0.1310	−0.3615	0.2998	33	0.0393	−0.4191	0.7318	55	0.0211	−0.4461	0.8367
12	0.0793	−0.4428	0.3928	34	0.0360	−0.4300	0.7419	56	0.0193	−0.4585	0.8414
13	0.0839	−0.4222	0.4166	35	0.0392	−0.4103	0.7426	57	0.0166	−0.4850	0.8472
14	0.0721	−0.4411	0.4563	36	0.0345	−0.4277	0.7549	58	0.0243	−0.4120	0.8373
15	0.0700	−0.4381	0.4825	37	0.0323	−0.4356	0.7627	59	0.0179	−0.4638	0.8492
16	0.0664	−0.4378	0.5096	38	0.0300	−0.4443	0.7704	60	0.0205	−0.4360	0.8478
17	0.0645	−0.4339	0.5321	39	0.0286	−0.4493	0.7759	61	0.0195	−0.4434	0.8505
18	0.0674	−0.4175	0.5449	40	0.0264	−0.4594	0.7842	62	0.0145	−0.4951	0.8605
19	0.0683	−0.4074	0.5598	41	0.0245	−0.4695	0.7904	63	0.0166	−0.4634	0.8581
20	0.0647	−0.4090	0.5783	42	0.0267	−0.4501	0.7905	64	0.0149	−0.4873	0.8628
21	0.0659	−0.3980	0.5912	43	0.0254	−0.4545	0.7966	65	0.0071	−0.6131	0.8773
22	0.0661	−0.3910	0.6039	44	0.0249	−0.4550	0.8004	66	0.0228	−0.4002	0.8536

**Table C2:** The coefficients for the fitting function of *μ* against $P_{f,ILS}$: $f_{\mu }\left(x\right)=p_{1}x^{p_{2}}+p_{3}$. The tolerable failure rate is $P_{f}^{tol}=0.001$.

*n*	$p_{1}$	$p_{2}$	$p_{3}$	*n*	$p_{1}$	$p_{2}$	$p_{3}$	*n*	$p_{1}$	$p_{2}$	$p_{3}$
1	0.0549	−0.4626	−0.1968	23	0.0347	−0.3933	0.5322	45	0.0095	−0.4982	0.7474
2	0.0507	−0.4739	−0.1450	24	0.0321	−0.3999	0.5500	46	0.0095	−0.4969	0.7525
3	0.0838	−0.3960	−0.1556	25	0.0318	−0.3958	0.5613	47	0.0085	−0.5058	0.7578
4	0.1343	−0.3225	−0.1755	26	0.0273	−0.4144	0.5805	48	0.0098	−0.4837	0.7602
5	0.1946	−0.2672	−0.1980	27	0.0261	−0.4147	0.5928	49	0.0105	−0.4706	0.7633
6	0.1876	−0.2651	−0.1429	28	0.0242	−0.4219	0.6072	50	0.0108	−0.4651	0.7673
7	0.1645	−0.2750	−0.0755	29	0.0226	−0.4288	0.6193	51	0.0072	−0.5210	0.7757
8	0.1751	−0.2605	−0.0404	30	0.0208	−0.4348	0.6309	52	0.0079	−0.5051	0.7767
9	0.1229	−0.3011	0.0634	31	0.0172	−0.4602	0.6431	53	0.0082	−0.4956	0.7819
10	0.1133	−0.3065	0.1151	32	0.0189	−0.4421	0.6524	54	0.0094	−0.4744	0.7840
11	0.0938	−0.3238	0.1795	33	0.0212	−0.4206	0.6574	55	0.0077	−0.5017	0.7885
12	0.0636	−0.3737	0.2505	34	0.0197	−0.4278	0.6673	56	0.0056	−0.5433	0.7956
13	0.0630	−0.3670	0.2833	35	0.0206	−0.4178	0.6716	57	0.0057	−0.5400	0.7998
14	0.0522	−0.3879	0.3263	36	0.0174	−0.4399	0.6852	58	0.0086	−0.4742	0.7975
15	0.0512	−0.3843	0.3543	37	0.0182	−0.4294	0.6901	59	0.0070	−0.4977	0.7998
16	0.0498	−0.3824	0.3789	38	0.0161	−0.4431	0.7004	60	0.0085	−0.4741	0.8039
17	0.0483	−0.3801	0.4054	39	0.0132	−0.4681	0.7071	61	0.0107	−0.4327	0.8016
18	0.0489	−0.3726	0.4257	40	0.0137	−0.4613	0.7155	62	0.0058	−0.5173	0.8121
19	0.0492	−0.3659	0.4450	41	0.0117	−0.4808	0.7232	63	0.0050	−0.5369	0.8181
20	0.0454	−0.3699	0.4690	42	0.0118	−0.4736	0.7286	64	0.0081	−0.4521	0.8137
21	0.0443	−0.3689	0.4880	43	0.0103	−0.4912	0.7351	65	0.0015	−0.7293	0.8205
22	0.0419	−0.3721	0.5072	44	0.0111	−0.4773	0.7402	66	0.0016	−0.7571	0.8317

### Appendix C.2. Coefficient Table for Fitting Functions of $P_{fix}$ against $P_{f}^{tol}$ in the Ratio Test

**Table C3:** The coefficients for the fitting function of $P_{fix}$ against $P_{f,ILS}$. The tolerable failure rate is $P_{f}^{tol}=0.01$. The function is shown in Equation (14). Note *$q_{4}=0.5035$.

*n*	$q_{1}$	$q_{2}$	$q_{3}$	*n*	$q_{1}$	$q_{2}$	$q_{3}$	*n*	$q_{1}$	$q_{2}$	$q_{3}$
1${}^{*}$	0.0225	0.0242	−0.3189	23	0.0218	0.0906	0.0200	45	0.0203	0.0476	0.0195
2	0.0081	−0.0139	0.0082	24	0.0180	0.0549	0.0168	46	0.0203	0.0423	0.0196
3	0.0132	0.0260	0.0127	25	0.0204	0.0574	0.0194	47	0.0194	0.0352	0.0188
4	0.0153	0.0252	0.0148	26	0.0228	0.0906	0.0211	48	0.0206	0.0412	0.0200
5	0.0176	0.0337	0.0170	27	0.0218	0.0833	0.0202	49	0.0229	0.0567	0.0220
6	0.0192	0.0482	0.0183	28	0.0198	0.0535	0.0189	50	0.0238	0.0605	0.0228
7	0.0176	0.0407	0.0169	29	0.0230	0.0902	0.0213	51	0.0175	0.0210	0.0172
8	0.0177	0.0384	0.0171	30	0.0207	0.0668	0.0195	52	0.0207	0.0432	0.0201
9	0.0186	0.0537	0.0176	31	0.0206	0.0521	0.0197	53	0.0193	0.0281	0.0189
10	0.0196	0.0630	0.0184	32	0.0191	0.0417	0.0184	54	0.0210	0.0358	0.0205
11	0.0205	0.0704	0.0192	33	0.0227	0.0634	0.0217	55	0.0213	0.0426	0.0206
12	0.0163	0.0644	0.0149	34	0.0235	0.0717	0.0223	56	0.0164	0.0137	0.0162
13	0.0139	0.0331	0.0132	35	0.0243	0.0709	0.0231	57	0.0160	0.0114	0.0159
14	0.0116	0.0203	0.0111	36	0.0252	0.0803	0.0239	58	0.0191	0.0316	0.0187
15	0.0121	0.0229	0.0115	37	0.0270	0.0964	0.0253	59	0.0165	0.0099	0.0164
16	0.0136	0.0342	0.0128	38	0.0273	0.1016	0.0255	60	0.0231	0.0528	0.0223
17	0.0164	0.0541	0.0153	39	0.0223	0.0605	0.0213	61	0.0224	0.0516	0.0216
18	0.0164	0.0443	0.0155	40	0.0252	0.0812	0.0239	62	0.0172	0.0197	0.0170
19	0.0163	0.0413	0.0154	41	0.0261	0.1038	0.0242	63	0.0151	0.0094	0.0150
20	0.0158	0.0395	0.0149	42	0.0216	0.0604	0.0205	64	0.0153	0.0065	0.0153
21	0.0193	0.0579	0.0183	43	0.0218	0.0560	0.0209	65	0.0106	−0.0266	0.0110
22	0.0210	0.0686	0.0198	44	0.0210	0.0542	0.0201	66	0.0148	−0.0067	0.0149

**Table C4:** The coefficients for the fitting function of $P_{fix}$ against $P_{f,ILS}$. The tolerable failure rate is $P_{f}^{tol}=0.001$. The function is shown in Equation (14). Note *$q_{4}=0.3811$.

*n*	$q_{1}$	$q_{2}$	$q_{3}$	*n*	$q_{1}$	$q_{2}$	$q_{3}$	*n*	$q_{1}$	$q_{2}$	$q_{3}$
1${}^{*}$	0.0229	0.0584	−0.3400	23	0.0036	0.0557	0.0032	45	0.0040	0.0395	0.0038
2	0.0012	−0.0065	0.0013	24	0.0037	0.0529	0.0035	46	0.0039	0.0312	0.0038
3	0.0016	0.0058	0.0016	25	0.0040	0.0588	0.0037	47	0.0039	0.0328	0.0038
4	0.0023	0.0216	0.0022	26	0.0039	0.0635	0.0036	48	0.0042	0.0413	0.0041
5	0.0029	0.0275	0.0028	27	0.0038	0.0590	0.0035	49	0.0038	0.0299	0.0037
6	0.0028	0.0280	0.0027	28	0.0037	0.0488	0.0035	50	0.0040	0.0293	0.0039
7	0.0025	0.0255	0.0024	29	0.0037	0.0528	0.0034	51	0.0041	0.0358	0.0039
8	0.0026	0.0252	0.0025	30	0.0039	0.0484	0.0036	52	0.0039	0.0306	0.0037
9	0.0024	0.0272	0.0022	31	0.0035	0.0363	0.0034	53	0.0037	0.0228	0.0036
10	0.0023	0.0263	0.0022	32	0.0040	0.0452	0.0037	54	0.0042	0.0296	0.0041
11	0.0025	0.0321	0.0023	33	0.0044	0.0509	0.0041	55	0.0043	0.0385	0.0041
12	0.0014	0.0076	0.0014	34	0.0049	0.0629	0.0046	56	0.0034	0.0227	0.0034
13	0.0016	0.0127	0.0015	35	0.0045	0.0459	0.0043	57	0.0030	0.0143	0.0030
14	0.0013	0.0061	0.0013	36	0.0053	0.0753	0.0049	58	0.0034	0.0176	0.0033
15	0.0014	0.0068	0.0014	37	0.0056	0.0787	0.0052	59	0.0037	0.0216	0.0037
16	0.0017	0.0169	0.0016	38	0.0062	0.0980	0.0057	60	0.0038	0.0271	0.0037
17	0.0019	0.0195	0.0018	39	0.0055	0.0763	0.0052	61	0.0035	0.0215	0.0034
18	0.0025	0.0328	0.0023	40	0.0056	0.0817	0.0052	62	0.0031	0.0155	0.0030
19	0.0026	0.0304	0.0024	41	0.0050	0.0710	0.0046	63	0.0024	0.0033	0.0024
20	0.0029	0.0401	0.0027	42	0.0045	0.0489	0.0043	64	0.0028	0.0080	0.0028
21	0.0032	0.0400	0.0030	43	0.0045	0.0493	0.0043	65	0.0029	0.0030	0.0029
22	0.0035	0.0504	0.0033	44	0.0042	0.0426	0.0040	66	0.0040	0.0266	0.0040
